# Transcriptome Analysis Revealed the Regulatory Mechanism of DIMBOA Affecting Early Somatic Embryogenesis in *Dimocarpus longan* Lour.

**DOI:** 10.3390/plants14030442

**Published:** 2025-02-03

**Authors:** Xiaoqiong Xu, Chunyu Zhang, Ning Tong, Xiaoyuan Lan, Jing Cui, Awais Muhammad, Zhilin Zhang, Zihao Zhang, Yukun Chen, Yuling Lin, Zhongxiong Lai

**Affiliations:** Institute of Horticultural Biotechnology, Fujian Agriculture and Forestry University, Fuzhou 350002, China; xuxq0921@163.com (X.X.); zcynhba@163.com (C.Z.); 17805953105@163.com (N.T.); littlelanyuan@163.com (X.L.); cuij200402@163.com (J.C.); awais9518@gmail.com (A.M.); hiqihai@163.com (Z.Z.); zhangzihao863@126.com (Z.Z.);

**Keywords:** *Dimocarpus longan* Lour., DIMBOA, somatic embryogenesis, RNA-seq, vitamin B_6_

## Abstract

*Dimocarpus longan* Lour. is an evergreen tree of the genus Longan in the Sapindaceae family, native to tropical and subtropical regions. Longan embryonic development is closely related to fruit set and fruit quality. An in-depth study of the mechanism of longan embryonic development could therefore contribute to the development of the longan industry. DIMBOA is the principal compound representing benzoxazinoids (BXs), and is closely linked to auxin biosynthesis and signal transduction. Auxin is one of the crucial hormones for inducing somatic embryogenesis (SE) in plants. Previous research has shown that DIMBOA promotes morphogenesis in the early somatic embryogenesis of longan, but the specific regulatory mechanism has not yet been clarified. To elucidate the molecular mechanism by which DIMBOA affects early somatic embryogenesis in longan, we chose longan embryogenic cultures grown under 0 mg/L DIMBOA as the control group (the check, CK), and longan embryogenic cultures grown under 0.1 mg/L DIMBOA as the treatment group (D) to be analyzed by transcriptomic sequencing. A total of 478 differentially expressed genes (DEGs) are detected in check vs. D, of which 193 are upregulated and 285 are downregulated. These DEGs are significantly enriched in the biosynthetic and metabolic functions of various substances such as vitamin B_6_ (VB_6_) biosynthesis, phenylpropanoid pathways, and carbohydrate metabolism. DIMBOA affects SE processes in longan via TFs, including MYB, ZF, bHLH, LBD, NAC, WRKY, etc. After DIMBOA treatment, the expression of most of the key genes for IAA synthesis was significantly downregulated, VB_6_ content was significantly reduced, and H_2_O_2_ content was significantly increased. Therefore, it is suggested that DIMBOA directly or indirectly affects the H_2_O_2_ content through the VB_6_ metabolic pathway, thereby regulating the endogenous IAA level to modulate the early SE morphogenesis of longan.

## 1. Introduction

*Dimocarpus longan* Lour. is an evergreen tree of the Longan genus in the Sapindaceae family, native to tropical and subtropical regions and widely planted in southern China and south-east Asia [1]. The development of longan embryos will seriously affect fruit set and quality, and in-depth research into the mechanism of longan embryo development is of great importance for the development of the longan industry [2]. However, research into the molecular mechanisms of longan embryo development in the natural state has many drawbacks, such as sampling difficulties, poor material synchronization, and uncontrollable developmental processes. Zhongxiong Lai [3,4] et al. established the longan somatic embryogenesis (SE) system, which has the advantages of higher synchronization, enhanced somatic embryogenesis frequency and advanced regeneration capacity, and this system can solve the problems encountered in the study of longan embryonic development well. Research conducted by Jiawei Wang’s team reveals that the transcriptional regulatory hierarchy network, which illustrates the embryogenesis of plant somatic cells, can establish a direct connection between cell totipotency transcription factors and early embryonic development. Consequently, the molecular study of longan somatic embryogenesis may offer a theoretical framework for embryonic development [5]. It is well known that most plant somatic cell embryos are induced by auxin [6] and that the process of somatic embryogenesis is regulated by the addition of different concentrations of auxin [7,8,9,10]. Previous research in our laboratory indicated that relatively high levels of endogenous indole-3-acetic acid (IAA) elevate the induction and maintenance of embryonic cytogenesis during longan SE, and that changes in endogenous IAA levels affect longan somatic embryo development [11]. Thus, endogenous IAA plays a crucial role during SE in longan. The longan somatic embryogenesis [12] system lays a solid foundation for further research into the mechanism of longan embryo development and, as a model system for somatic embryogenesis in woody plants, provides a theoretical basis for the study of somatic embryogenesis in woody plants.

DIMBOA (2,4-dihydroxy-7-methoxy-2H-1,4-benzoxazine-3(4H)-one) is the main substance representing the benzoxazinoid (BX) compounds. Based on studies of BX compounds in grasses, we know that DIMBOA was the major resistance and chemosensory substance in maize, with effects on plant root growth and involvement in auxin metabolism. DIMBOA is also a major hydroxamic acid, generally present in plants in the form of DIMBOA-Glc [13]. Callus tissue from wheat, barley, and maize contained DIMBOA-Glc [14,15,16]. Among them, in barley callus, the DIMBOA-Glc content of regenerable callus was higher than that of non-regenerable callus, and the regenerative capacity of callus was found to be closely linked to DIMBOA-Glc [15]. It was found that exogenous application of DIMBOA could significantly enhance the germination rate, germination potential, radicle length, embryo length, and seedling height of alfalfa seeds [17], which play a key role in plant growth and development. In addition, DIMBOA and MBOA (6-methoxybenzoxazolin-3-one) had the property of modifying the binding affinity of growth hormones to receptor sites and were capable of modifying the growth pattern of oats, timothy, pigweed, and peas as a result of this attribute [18,19,20,21]. DIMBOA also promoted the auxin-induced elongation of maize germ sheath segments [22]. In addition, DIMBOA has previously been identified as a compound that regulates the response of maize germ sheaths to phototropism [23]. It has also been suggested that DIMBOA and MBOA are auxin inhibitors that bind to membrane components and proteins [24]. Previous studies have shown that exogenous DIMBOA indirectly promotes early somatic embryogenesis morphogenesis in longan by regulating endogenous auxin biosynthesis and transport in longan embryogenic cultures [25]. Studies on the phototropism of maize embryo sheaths have revealed that phototaxis induced the production of BX compounds, leading to an increase in hydrogen peroxide (H_2_O_2_) content on the light-exposed side, thereby enhancing cell wall rigidity and inhibiting embryo sheath growth on the light-exposed side of the sheath [26]. Exogenous treatment of oats with DIMBOA promoted H_2_O_2_ synthesis, acting as a growth inhibitor [20]. In recent years, it has been discovered that H_2_O_2_ can regulate auxin accumulation and redistribution by modulating auxin polar transport [27], and that H_2_O_2_ regulates plant growth and development mainly by inhibiting auxin signaling in plants [28]. Therefore, it is highly promising that DIMBOA affects auxin biosynthesis and signal transduction processes by influencing H_2_O_2_ synthesis. Auxin biosynthesis and signal transduction play an essential role in plant somatic embryogenesis. Therefore, it is very important to study the particular regulatory mechanisms of DIMBOA during somatic embryogenesis in plants.

Transcriptomic data of numerous plant species have revealed regulatory mechanisms during embryogenesis such as in *Arabidopsis thaliana* [29,30], *Camellia sinensis* [31], *Coffea arabica* [32], *Hevea brasiliensis* [33], *Dimocarpus longan* [2], and *Tilia amurensis* [34]. In addition, Rongzhu Chen analyzed the metabolic pathways associated with DEGs during the early somatic embryogenesis of longan induced by 5-azac by transcriptome sequencing, and further elucidated the DNA methylation mechanism during the somatic embryogenesis of longan [35]. Barbara [29] et al. analyzed the transcriptome data of the *Arabidopsis thaliana* somatic embryogenesis induction process after treatment with the histone deacetylase inhibitor trichostatin A (TSA) and found that TSA upregulated transcription factors (TFs) that play a key role in auxin-induced SE and most stress-related genes, providing new insights into the epigenetic network of embryogenesis transformation in plant somatic cells. Previous studies have shown that treatment with exogenous DIMBOA promotes the morphogenesis of early somatic embryogenesis in longan [25]. However, specific regulatory mechanisms for the effects of DIMBOA treatment on somatic embryogenesis in longan have not been reported. In order to further explore the molecular mechanism of exogenous DIMBOA treatment on the early somatic embryogenesis of longan, the embryonic callus of ‘Honghezi’ longan cultured in our laboratory was used as the material, and different concentrations of DIMBOA were added to the culture to study the changes of H_2_O_2_ content and vitamin B_6_ (VB_6_) content. In addition, transcriptome sequencing was carried out, and the DEGs were compared and analyzed through the transcriptome sequencing data, and the metabolic pathways and TFs related to the DEGs were analyzed, which laid a foundation for further elucidation of the molecular mechanism of DIMBOA regulating the somatic embryogenesis of longan.

## 2. Results

### 2.1. Transcriptome Sequencing Quality Assessment

**Figure 1:**
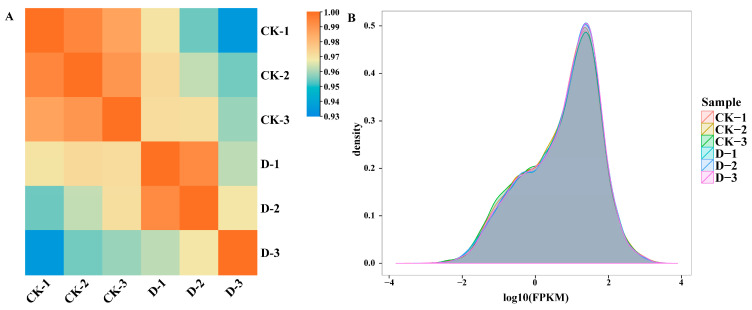
Gene transcription abundances of samples at different concentrations treated with exogenous DIMBOA. (**A**) Pearson correlation analysis between samples based on the FPKM values of the genes; the color represents the size of the r value. (**B**) Comparison of the density distribution of the FPKM values of each sample; the different-colored curves in the figure represent different samples, the horizontal coordinates indicate the logarithmic values of the FPKM of the corresponding samples, and the vertical coordinates indicate the probability densities. CK is an abbreviation for the check.

Previous experimental results indicated that treatment with 0.1 mg/L DIMBOA significantly promoted cell proliferation and the formation of globular embryos (GEs) during the early SE process [25]. To explore the molecular mechanisms underlying DIMBOA-induced promotion of somatic embryogenesis in longan, this study collected longan embryogenic cultures treated with 0.1 mg/L DIMBOA (denoted as D) and the control group (the check denoted as CK), and constructed six mRNA libraries. After quality control of the raw transcriptome sequencing data, a total of 41.60 Gb of valid data was obtained, with each sample providing more than 6.17 Gb of effective data. The GC content of the clean reads ranged from 43.80% to 44.13%, and the percentage of Q30 bases was over 96.07% for all samples. After aligning the effective data from all samples to the reference genome, the transcriptome data covered more than 95.43% of the reference genome, indicating high coverage and reliability of the sequencing data, making them suitable for subsequent experimental analysis. Pearson correlation coefficients were used to evaluate the biological reproducibility of the transcriptome data. The results showed that the biological replicates within the same group had a higher correlation, with r > 0.93 (Figure 1A). In addition, the FPKM value distribution for the samples within each group was similar (Figure 1B), depicting good reproducibility of the transcriptome sequencing results for the three biological replicates. Based on the alignment results, 2268 new genes were speculated, of which 929 were functionally annotated.

### 2.2. Data Analysis and Identification of Differentially Expressed Genes Under DIMBOA Treatment

**Figure 2:**
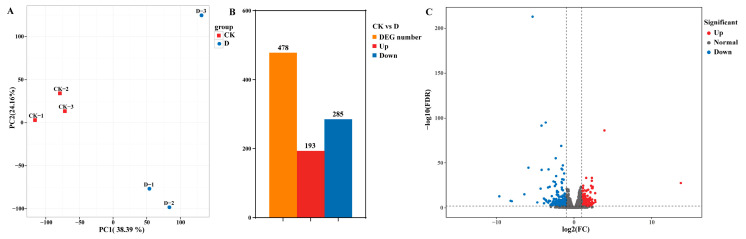
DEGs in embryogenic cultures of *D. longan* between the CK and DIMBOA-treated groups. (**A**) Correlation among samples in the CK and DIMBOA groups. (**B**) Number of significantly upregulated and downregulated genes in different groups. (**C**) Volcano plot of identified genes including upregulated and downregulated genes in the RNA-seq. CK is an abbreviation for the check.

PCA (Principal Component Analysis) results showed a clear separation between the check and DIMBOA-treated groups (Figure 2A), indicating that the gene expression patterns in longan embryogenic cultures were significantly altered following DIMBOA treatment. DEGs were selected based on the criteria of |log_2_ (fold change)| > 1 and FDR < 0.01. We identified a total of 478 DEGs in this study, out of which 193 genes were upregulated and 285 genes downregulated (Figure 2B,C). The results suggest that after DIMBOA treatment of longan embryogenic callus (EC), the number of downregulated genes was higher than the number of upregulated genes.

**Figure 3:**
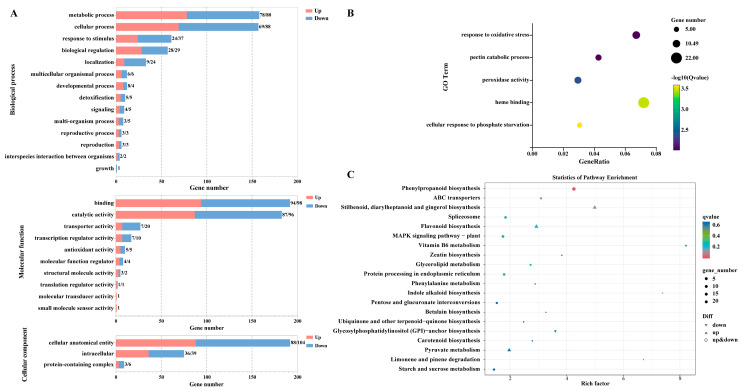
GO enrichment and KEGG enrichment analysis for the DEGs in the embryogenic cultures of *D. longan* between the CK and DIMBOA-treated groups. (**A**) GO enrichment terms of DEGs in level 1 GO terms and level 2 GO terms. (**B**) Significantly differential GO terms. (**C**) Top 20 KEGG enrichment pathways of CK vs. D. CK is an abbreviation for the check.

To further investigate the functions of the DEGs, GO (Gene Ontology) analysis was performed (Figure 3A, Supplementary Table S1). The results showed that the DEGs were primarily associated with biological processes (BPs), molecular functions (MFs), and cellular components (CCs). After DIMBOA treatment of longan ECs, in the BP category, DEGs were enriched in 14 GO terms, mainly focusing on metabolic process, cellular process, response to stimulus, biological regulation, and localization. In the MF category, DEGs were enriched in 10 GO terms, with a primary focus on binding, catalytic activity, and transporter activity. In the CC category, DEGs were enriched in three GO terms, named cellular anatomical entity, intracellular, and protein-containing complex. Moreover, after DIMBOA treatment, five specific GO terms exhibited significant changes: cellular response to phosphate starvation, heme binding, peroxidase activity, pectin catabolic process, and response to oxidative stress (Figure 3B). These findings suggest that DIMBOA treatment significantly affects various biological functions and processes in longan embryogenic cultures, especially those affiliated with metabolism, stress response, and enzymatic activity.

### 2.3. KEGG Enrichment Analysis

As shown in Supplementary Table S2, 276 DEGs were found to be enriched in 5 KEGG Class A pathways and 19 KEGG Class B pathways. Additionally, among the top 20 KEGG pathways, metabolic pathways were predominantly described (Figure 3C), particularly those connected to cofactors and vitamins, carbohydrates, terpenoids and polyketides, and other secondary metabolites. The present study is centralized around the most significantly enriched pathways, including the VB_6_ metabolic pathway, phenylpropanoid-related pathway, and carbohydrate metabolic pathways. These pathways are of particular interest due to their role in various metabolic and stress response processes in longan embryogenic cultures following DIMBOA treatment.

### 2.4. DEGs Involved in Vitamin B_6_ Biosynthesis Pathway in Longan Embryogenic Cultures

**Figure 4:**
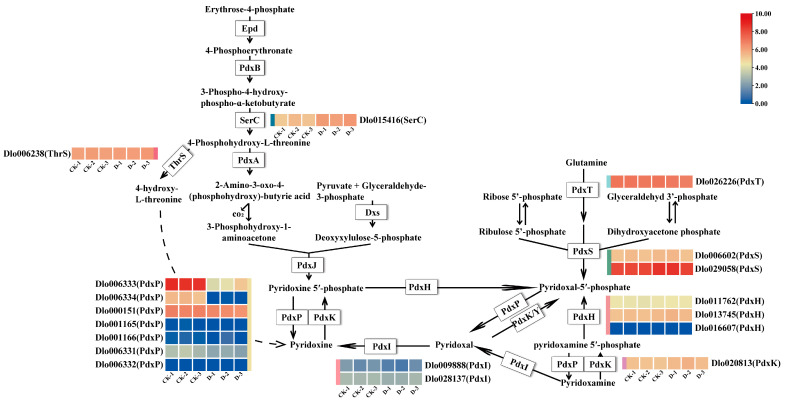
Expression profiles of key structural genes in the VB_6_ biosynthesis pathway of *D. longan*. Structural genes in each catalytic step are labeled in white boxes. Enzymes: Dxs, 1-deoxyxylulose 5-phosphate synthase; Epd, erythrose 4-phosphate dehydrogenase; PdxA, 4-phosphohydroxy-L-threonine dehydrogenase; PdxB, 4-phosphoerythronate dehydrogenase; PdxH, PNP oxidase; PdxJ, PNP synthase; PdxK, PN, PL, and PM kinase; PdxP, pyridoxal phosphate phosphatase; PdxS, PLP synthase subunit; PdxT, glutaminase subunit (PdxS and PdxT form the PLP synthase complex); PdxY, pyridoxal kinase; pdxI, pyridoxine 4-dehydrogenase; ThrS, threonine synthase; SerC, 3-phosphoserine aminotransferase. Gradient colors for each gene represent gene expression levels [normalized using log2 transformation (FPKM + 1). CK is an abbreviation for the check.

VB_6_ refers collectively to pyridoxal (PL), pyridoxine (PN), pyridoxamine (PM) and their corresponding phosphate esters. Their catalytically active forms are pyridoxal 5′-phosphate (PLP), pyridoxine 5′-phosphate (PNP) and pyridoxamine 5′-phosphate (PMP). The VB_6_ metabolism pathway was enriched in the check vs. D [36]. Based on the transcriptome sequencing annotation results, a total of 18 genes in longan were annotated to the VB_6_ metabolic pathway. Using the gene annotation results and the expression levels of relevant genes, a pathway diagram of the longan VB_6_ biosynthesis pathway was constructed (Figure 4, Supplementary Table S3). This pathway map highlights the key genes engaged in the biosynthesis of VB_6_ in longan and provides insights into the molecular mechanisms underlying the regulation of VB_6_ metabolism in response to DIMBOA treatment. Our findings have shown that *PdxP* (*pyridoxal phosphate phosphatase*) (Dlo001166, Dlo001165, Dlo006332 and Dlo016607) was expressed at very low or no levels in the VB_6_ metabolism pathway. *PdxP* (Dlo006333, Dlo006334, Dlo000151, Dlo001166, and Dlo006331), *PdxI* (*pyridoxine 4-dehydrogenase*) (Dlo009888 and Dlo028137), and *PdxT* (*pyridoxal 5′-phosphate synthase*) (Dlo026226) were downregulated in the VB_6_ metabolism pathway. *ThrS* (threonine synthase) (Dlo006238), *PdxS* (*Pyridoxal 5′-phosphate synthase*) (Dlo029058 and Dlo006602), *PdxH* (*Pyridoxamine 5′-phosphate oxidase*) (Dlo013745 and Dlo011762), *SerC* (*3-phosphoserine aminotransferase*) (Dlo015416), and *PdxK* (*Pyridoxal kinase*) (Dlo020813) were upregulated in the VB_6_ metabolism pathway. Among them, *PdxP* (Dlo006333 and Dlo006334) was significantly downregulated in VB_6_ metabolism. In general, all DEGs showed downregulated expression in the VB_6_ metabolism pathway, which may be associated with VB_6_ accumulation.

### 2.5. DEGs Involved in Phenylpropanoid Metabolism-Related Pathways in Longan Embryogenic Cultures

**Figure 5:**
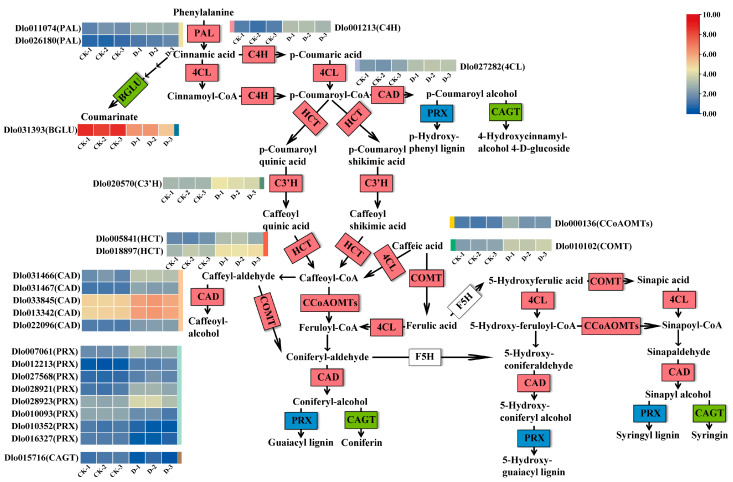
Expression profiles of differentially expressed genes in the phenylpropanoid biosynthesis pathway of *D. longan*. The structural genes in each catalytic step are labeled in the box, with red indicating significant upregulation, green indicating significant downregulation, and blue indicating both significant upregulation and significant downregulation. Enzymes: PAL, phenylalanine ammonia lyase; C4H, cinnamate 4-hydroxylase; 4CL, 4-coumarate-CoA ligase; BGLU, beta-glucosidase; HCT, hydroxycinnamoyl-CoA shikimate transferase; CAD, cinnamyl alcohol dehydrogenase; COMT, caffeic acid 3-O-methyltransferase; CCoAOMTs, caffeoyl CoA O-methyltransferases; PRX, peroxidases; CAGT, coniferyl alcohol glucosyltransferase; C3′H, cinnamoyl ester 3′-hydroxylase. Gradient colors for each gene represent gene expression levels [normalized using log2 transformation (FPKM + 1). CK is an abbreviation for the check.

The phenylpropanoid biosynthesis, stilbenoid, diarylheptanoid and gingerol biosynthesis, and flavonoid biosynthesis pathways were enriched in the check vs. D. Flavonoids, stilbenoid, diarylheptanoid, and gingerol are synthesized through the phenylpropanoid pathway, transforming phenylalanine into 4-coumaroyl-CoA, which finally enters the flavonoid and stilbenoid, diarylheptanoid and gingerol biosynthesis pathway (Figure 5, Supplementary Table S3). Prominently, DIMBOA treatment resulted in the presence of 24 DEGs in the phenylpropanoid biosynthesis pathway, including 5 downregulated DEGs and 19 upregulated DEGs. The five downregulated DEGs are *PRX* (*Peroxidases*) (Dlo010093, Dlo010352 and Dlo016327), *CAGT* (*coniferyl alcohol glucosyltransferase*) (Dlo015716), and *BGLU* (*beta-glucosidase*) (Dlo031393). In addition, there were five DEGs in the stilbenoid, diarylheptanoid and gingerol biosynthesis and flavonoid biosynthesis pathways after DIMBOA treatment. It is worth noting that *CCoAOMT* (*caffeoyl-CoA O-methyltransferase*) (Dlo000136), *C4H* (*Cinnamate 4-hydroxylase*) (Dlo001213), *HCT* (*Hydroxycinnamoyl-CoA shikimate transferase*) (Dlo005841 and Dlo018897), and *C3′H* (*cinnamoyl ester 3′-hydroxylase*) (Dlo020570) were enriched in phenylpropanoid, flavonoid, stilbenoid, diarylheptanoid, and gingerol biosynthesis pathways, and all of them were significantly upregulated.

### 2.6. DEGs Involved in Carbohydrate Metabolism in Longan Embryogenic Cultures

**Figure 6:**
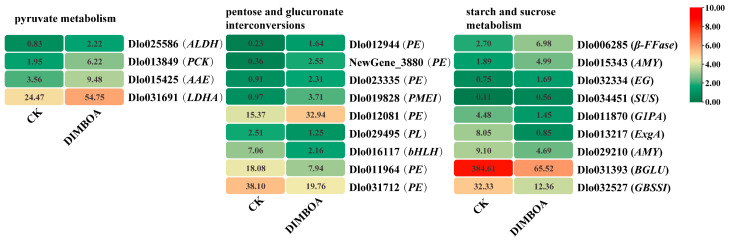
Expression of DEGs in carbohydrate metabolism pathways. CK is an abbreviation for the check.

In total, three pathways of carbohydrate metabolism were enriched: pyruvate metabolism, pentose and glucuronate interconversions, and starch and sucrose metabolism. After DIMBOA treatment, pyruvate metabolism was significantly changed as compared to the check group. Based on the transcriptome sequencing annotation results, a total of 150 genes in longan were annotated to the pyruvate metabolic pathway, including 4 DEGs. Among these, 49 genes exhibited low or no expression in this pathway. Additionally, 38 genes were found to be downregulated, while 63 genes showed upregulation in the pyruvate metabolic pathway. Among them, *ALDH* (*Aldehyde dehydrogenase*) (Dlo025586), *PCK* (*Phosphoenolpyruvate carboxykinase*) (Dlo013849), *AAE* (*acyl-activating enzyme*) (Dlo015425), and *LDHA* (*Lactate dehydrogenase A*) (Dlo031691) were significantly upregulated in the pyruvate metabolism pathway. Consequently, all DEGs in the pyruvate metabolic pathway exhibited upregulated expression, which may influence the accumulation of pyruvate in longan embryogenic cultures. These findings indicate significant transcriptional alterations in genes associated with pyruvate metabolism in response to DIMBOA treatment, which may contribute to the observed physiological and biochemical responses in longan embryogenic cultures. In addition, a total of 428 genes in longan were annotated to the pentose and glucuronate interconversion pathway, among which 272 genes showed very low or no expression. Of the remaining genes, 77 were downregulated, and 79 were upregulated. Notably, in this pathway, four DEGs were downregulated, and five DEGs were upregulated following DIMBOA treatment. In the starch and sucrose metabolism pathway, 510 genes were annotated, with 173 showing very low or no expression. Among the expressed genes, 140 were downregulated, and 197 were upregulated. It is noteworthy that the starch and sucrose metabolism pathway included four upregulated DEGs and six downregulated DEGs after DIMBOA treatment. These findings propose significant regulatory effects of DIMBOA on carbohydrate metabolism pathways in longan embryogenic cultures (Figure 6, Supplementary Table S3).

### 2.7. Response of Differentially Expressed TFs to DIMBOA

**Figure 7:**
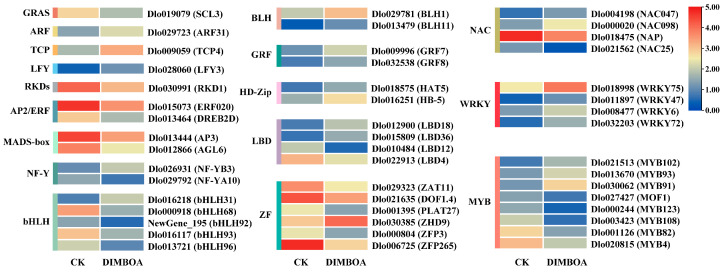
DEGs encoded for major transcription factor families. CK is an abbreviation for the check.

TFs are a class of proteins that exert their biological functions by regulating the transcription of target genes, and play a key role in somatic embryogenesis in plants [37]. We analyzed the expression patterns of differentially expressed TFs following DIMBOA treatment and identified a total of 48 TFs belonging to 17 distinct families (Figure 7, Supplementary Table S3). The major transcription factor families include MYB (eight genes), ZF (zinc finger, six genes), bHLH (five genes), LBD (four genes), NAC (four genes), and WRKY (four genes), all showing varied regulation of DEGs. Additionally, six TF families (AP2/ERF, BLH, GRF, HD-Zip, MADS-box, and NF-Y) each contained two differentially expressed genes, while four TF families (ARF, GRAS, LFY, and RKDs) had only one differentially expressed gene. Under DIMBOA treatment, MYB, ZF, bHLH, LBD, NAC, and NF-Y families exhibited both upregulated and downregulated genes, suggesting that these TF families may act as both positive and negative regulators. Contrarily, the TFs from the AP2/ERF, MADS-box, GRAS, and RKD families were exclusively downregulated, whereas members of the WRKY, BLH, HD-Zip, ARF, GRF, LFY, and TCP families were significantly upregulated. According to the transcriptome annotation file (see Supplementary Table S3), nine differentially expressed transcription factors were mapped to the KEGG pathway. Among them, Dlo013721 (bHLH96), Dlo011897 (WRKY47), Dlo008477 (WRKY6), and Dlo032203 (WRKY72) were annotated as involved in the MAPK signaling pathway of plants. Dlo019079 (SCL3) and Dlo027427 (MOF1) were annotated to be involved in plant hormone signal transduction. Dlo016117 (bHLH93), Dlo018998 (WRKY75), and Dlo030385 (ZHD9) were annotated as involved in pentose and glucuronate interconversions, spliceosome, and betalain biosynthesis, respectively. These results express a complex regulatory network involving multiple TF families that mediate the transcriptional response to DIMBOA treatment in longan embryogenic cultures.

### 2.8. Alternative Splicing Analysis

**Figure 8:**
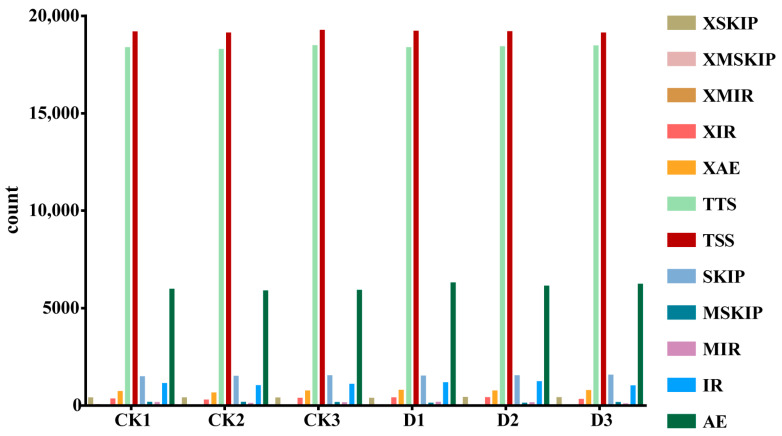
Alternative splicing events in longan embryogenic cultures after DIMBOA treatment. TSS, transcript start site. TTS, transcript end site. IR, intron retention. AE, alternative exon end. SKIP, skipped exon. XIR, IR-like. XAE, AE-like. XSKIP, SKIP-like. MIR, multi-IR. XMIR, MIR-like. MSKIP, multi-exon SKIP. XMSKIP, MSKIP-like. CK is an abbreviation for the check.

Various RNA splice isoforms can be generated through different splicing patterns or splice sites, resulting in the translation of diverse proteins and contributing to biological trait diversity. This post-transcriptional mRNA processing is referred to as alternative splicing (AS). AS is widely observed in eukaryotes and serves as a critical mechanism for regulating gene expression and enhancing protein diversity. There are five types of AS events: alternative exon end (AE), intron retention (IR), skipped exon (SKIP), transcript start site (TSS), and transcript end site (TTS) (Figure 8, Supplementary Table S4). Classification and quantitative analysis of AS events after DIMBOA treatment were performed (Figure 8). The results revealed that TSS and TTS accounted for the highest proportions of AS events, followed by AE and SKIP, indicating that DIMBOA treatment significantly influences alternative splicing activity in longan embryogenic cultures.

### 2.9. Expression Analysis of Key Genes of Auxin Biosynthesis Under Different Concentrations of DIMBOA

**Figure 9:**
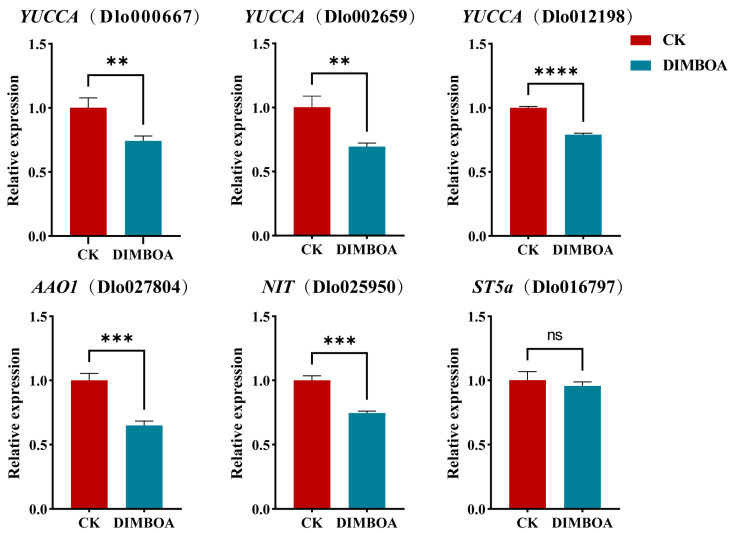
Relative expression of genes related to IAA biosynthesis. ns indicates no significant difference, and an asterisk indicates a significant difference (** *p* ≤ 0.01, *** *p* ≤ 0.001 and **** *p* ≤ 0.0001). CK is an abbreviation for the check.

Previous studies have shown that exogenous DIMBOA treatment affects the accumulation of endogenous IAA in longan EC [25]. To further enquire whether exogenous DIMBOA influences endogenous auxin biosynthesis, our laboratory conducted transcriptome analyses based on second-generation sequencing of early SE in longan. The results indicated that key genes in the IAA biosynthesis pathway, including *YUCCA* (Dlo_013505.1, Dlo_027234.1, Dlo_024140.1), *AAO1* (Dlo_016663.1), *NIT* (Dlo_032471.1), and *ST5a* (Dlo_007467.1), were significantly expressed during early SE in longan. In this study, based on the third-generation genome of longan, local BLAST analyses were performed to study the expression of the homologous genes *YUCCA* (Dlo000667, Dlo002659, Dlo012198), *AAO1* (Dlo027804), *NIT* (Dlo025950), and *ST5a* (Dlo016797) under exogenous DIMBOA treatment. The results (Figure 9) showed that after DIMBOA treatment, the expression of key genes in the IAA biosynthesis pathway was generally downregulated in comparison to the check. Notably, all genes except *ST5a* (Dlo016797) were significantly downregulated. These findings suggest that DIMBOA influences the accumulation of endogenous IAA by regulating the expression of key genes involved in IAA biosynthesis.

### 2.10. Determination of H_2_O_2_ Content and Vitamin B_6_ Content

**Figure 10:**
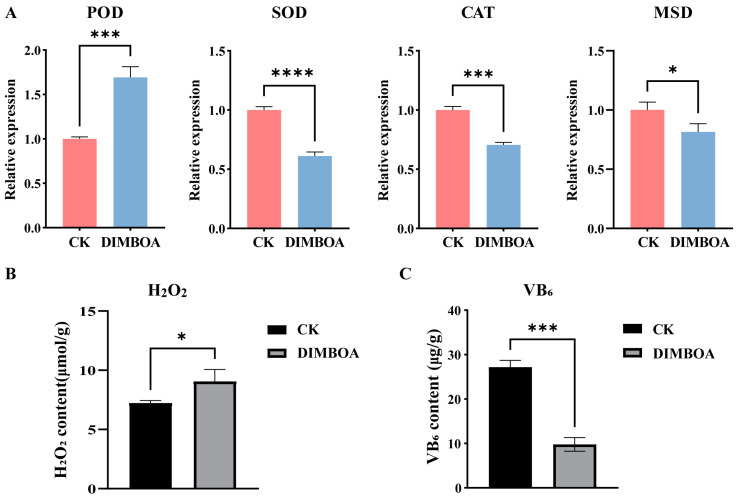
VB_6_ content, H_2_O_2_ content, and the expression of ROS-related genes. (**A**) Expression level of ROS-related genes; (**B**) H_2_O_2_ content; (**C**) VB_6_ content. An asterisk indicates distinctiveness (* *p* ≤ 0.05, *** *p* ≤ 0.001 and **** *p* ≤ 0.0001). CK is an abbreviation for the check.

The expression patterns of reactive oxygen species (ROS)-related genes, including *POD*, *CAT*, *SOD*, and *MSD*, were analyzed (Figure 10A). As shown in Figure 10A, compared to the check, the expression levels of all genes except *POD* were significantly downregulated under DIMBOA treatment. Notably, under DIMBOA treatment, *POD* transcription was significantly regulated, with its expression level showing a 1.7-fold increase. This suggests that *POD* may play a crucial role in the DIMBOA-mediated regulation of early SE in longan.

Previous studies have demonstrated that exogenous DIMBOA treatment promotes H_2_O_2_ synthesis, and H_2_O_2_ can influence the accumulation and redistribution of auxin. To explore whether DIMBOA indirectly affects the early SE process in longan by regulating H_2_O_2_ levels, we analyzed the changes in H_2_O_2_ content in longan embryogenic cultures after DIMBOA treatment. The results (Figure 10B) showed that under DIMBOA treatment, H_2_O_2_ content was significantly increased. These findings indicate that DIMBOA affects H_2_O_2_ accumulation, suggesting a potential regulatory role of H_2_O_2_ in the DIMBOA-mediated promotion of early SE in longan.

In this study, we found that the VB_6_ metabolic pathway was significantly enriched during DIMBOA-induced SE in longan. Therefore, we measured the VB_6_ content in longan embryogenic cultures (Figure 10C). The results showed that compared to the control group, VB_6_ content in the treatment group was significantly reduced.

### 2.11. qRT-PCR Analysis of DEGs

**Figure 11:**
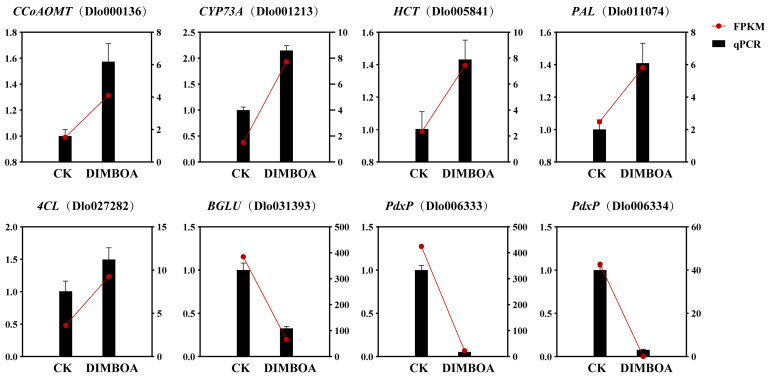
qRT-PCR analysis of DEGs. CK is an abbreviation for the check.

To validate the reliability of transcriptome expression data, eight genes were randomly selected from DEGs in key metabolic pathways for qRT-PCR analysis, including *CCoAOMT* (Dlo000136), *C4H* (Dlo001213), *HCT* (Dlo005841), *PAL* (Dlo011074), *4CL* (Dlo027282), *BGLU* (Dlo031393), and *PdxP* (Dlo006333 and Dlo006334). The qRT-PCR results showed that the expression patterns of these eight genes were generally consistent with the transcriptome data (Figure 11), confirming the high reliability of the transcriptome analysis.

## 3. Discussion

### 3.1. DIMBOA May Regulate Early SE of Longan via Various Metabolic Pathways

Phenylpropanoid metabolism is one of the most important metabolisms in plants, which gives rise to metabolites including lignin, flavonoids, lignans, phenylpropanoid esters, hydroxycinnamic acid amides, and sporopollenin [38]. The phenylpropanoid pathway lies at the nexus of plant growth and development, structural support, and responses to both biotic and abiotic stresses. External stimuli are major driving forces in inducing SE in plants [39]. This pathway plays a pivotal role in SE, root formation, and auxin metabolism [40,41,42]. For instance, Carol A. [43] et al. used proteomics and metabolomics to compare embryogenic and non-embryogenic cultures in avocado, revealing higher phenylpropanoid pathway activity in EC. In *Quercus petraea*, applying the phenylpropanoid biosynthesis inhibitor 2-aminoindan-2-phosphonic acid reduced lignin content and altered the proportions of different somatic embryo types [44]. The phenylpropanoid pathway has been linked to the auxin response in plants. Recent studies have shown that phenylpropanoid biosynthesis, especially trans-cinnamic acid derivatives, enhances auxin signaling and promotes auxin-dependent leaf expansion in *Arabidopsis* [45]. Flavonoid pathway intermediates like naringenin inhibit auxin transport [46], while cinnamic acid interferes with auxin efflux [47]. In cotton, flavonoids specifically accumulate during somatic embryo development [48], and genes associated with flavonoid biosynthesis are upregulated in cacao somatic embryos [49]. Conversely, in *Larix kaempferi*, phenylpropanoid and flavonoid biosynthesis genes (*C4H*, *CCOAOMT*, *HCT*) exhibit higher expression in NEC than in EC, indicating a complex relationship between phenylpropanoid compound accumulation and SE [50]. In the current study, pathways related to phenylpropanoid biosynthesis, stilbenoid, diarylheptanoid and gingerol biosynthesis, and flavonoid biosynthesis were significantly enriched during DIMBOA-induced early SE in longan. Most phenylpropanoid pathway-related DEGs, such as *PAL*, *4CL*, *C3′H*, *C4H*, *CAD*, *CCoAOMT*, *COMT*, and *HCT* were upregulated. Similarly, in *Korean pine* SE cell lines, *PAL*, flavonoid 3′-monooxygenase, *CCoAOMT*, and *2OGD*-related genes were highly expressed [51]. These findings suggest that DIMBOA may regulate early somatic embryo formation in longan through the phenylpropanoid pathway, emphasizing its role in modulating auxin-related processes and the accumulation of phenylpropanoid-derived compounds.

Carbohydrates and plant growth regulators are important variables affecting SE in plants. In addition to being sources of carbon and energy, carbohydrates also act as osmotic agents, protective agents, and crucial signaling molecules. Studies have shown that carbohydrate metabolism regulates somatic embryogenesis in *Araucaria angustifolia* [52]. In the present study, three carbohydrate metabolism pathways were significantly enriched following DIMBOA treatment: the pyruvate metabolism pathway, starch and sucrose metabolism pathway, and pentose and glucuronate interconversions pathway. These pathways are crucial for energy production, biosynthesis of key metabolites, and the regulation of osmotic pressure within cells. Given these findings, it is highly likely that DIMBOA influences the early stages of SE in longan by modulating carbohydrate metabolism, which may affect energy balance, osmotic regulation, and signaling during embryo development. This suggests a potential link between carbohydrate metabolism and the regulation of SE, further supporting the role of DIMBOA in influencing early somatic embryo formation.

Pyruvate, a key metabolite derived from pyruvic acid, plays a crucial role in both glycolysis and mitochondrial respiration, which are fundamental processes in plant carbon metabolism. In the present study, KEGG enrichment analysis showed significant clustering of genes related to pyruvate metabolism in longan embryogenic cultures after DIMBOA treatment. In cotton SE cultures, transcriptome analysis has revealed differential expression of pyruvate metabolism-related genes between non-embryogenic callus and primary embryogenic callus [53]. Additionally, significant variations in pyruvate metabolism were observed in highly embryogenic genotypes of cotton [54]. Proteomic analysis using iTRAQ has also identified key genes/proteins related to pyruvate metabolism [55], demonstrating that pyruvate and arginine metabolism are crucial for cell division and differentiation during SE [56]. Similarly, in maize cell lines, differential gene enrichment analysis highlighted pyruvate metabolism, plant hormone synthesis, and fatty acid synthesis as the most affected pathways, emphasizing the conserved and essential role of pyruvate metabolism in SE [57]. In this study, genes involved in pyruvate metabolism, including *ALDH*, *PCK*, *AAE*, and *LDHA,* were significantly upregulated. This upregulation likely promotes pyruvate metabolism, leading to a reduction in pyruvate content. These results suggest that DIMBOA may play a key role in regulating pyruvate metabolism during the somatic embryogenesis process in longan, highlighting the importance of this metabolic pathway in SE.

Sucrose is the most commonly used carbon source in plant tissue culture. During conifer embryonic development, storage compounds such as lipids, starch, and proteins are considered important features of embryo development [58]. The exogenous application of glucose, sucrose, and galactose has been shown to promote early maturation of somatic embryos in *Abies alba* [59], highlighting the role of sucrose in inducing, proliferating, and maturing plant SE. Moreover, previous studies have indicated that growth regulation in plant embryo tissues involves both ABA (abscisic acid) and sugar metabolism [60]. In Norway spruce embryo cultures, higher enzyme activity during proliferation and early maturation stages was found to accelerate sucrose hydrolysis in the medium [61]. Interestingly, the enzyme activity of invertase decreases as SE develops [62]. In the present study, two DEGs (Dlo001138 and Dlo006285) were found to encode invertase enzymes, with one DEG showing significantly upregulated expression and the other showing downregulation. This suggests that DIMBOA may regulate sucrose content by affecting the activity of these enzymes, thereby influencing endogenous carbohydrate metabolism. The aggregation of starch during the maturation stage is associated with the formation of early somatic embryos and the responsiveness of cell lines [52]. Starch metabolism is central to the embryo development process in *Araucaria angustifolia* [63]. Therefore, regulating starch metabolism could be a strategy to enhance somatic embryogenesis in plants [63]. In the starch biosynthesis pathway, *GBSS* is responsible for starch synthesis, while *AMY* is involved in starch degradation. In this study, *GBSSI* (Dlo032527) and *AMY* (Dlo029210) were significantly downregulated, whereas *AMY* (Dlo015343) was significantly upregulated. These findings suggest that DIMBOA may modulate the expression levels of related genes, thereby affecting both starch synthesis and degradation processes during SE in longan.

### 3.2. TFs in Response to Exogenous DIMBOA Treatment Are Involved in Regulating Early SE Processes in Longan

TFs play critical regulatory roles in plant embryo development. Numerous studies have revealed complex transcriptional regulatory networks during EC formation and the SE process [64,65]. In this study, following DIMBOA treatment, 17 TF families exhibited DEGs, including MYB, ZF, bHLH, LBD, NAC, WRKY, AP2/ERF, BLH, GRF, HD-Zip, MADS-box, NF-Y, ARF, GRAS, LFY, RKDs, and TCP. The major families, such as MYB, ZF, bHLH, LBD, NAC, and WRKY, displayed varying degrees of regulation on DEGs. These TFs play significant roles in responding to various stresses. For instance, MYB is involved in plant development, growth, hormone signaling, and flavonoid biosynthesis. The AP2/ERF family plays a crucial role in cell proliferation and embryogenesis [66]. WRKY TFs respond to abiotic stresses [67,68], while bHLH regulates lignin biosynthesis [64]. During early somatic embryogenesis in *Ginkgo biloba*, many MYB TFs are expressed at the EC stage [69]. In *Arabidopsis*, overexpression of *PGA37/MYB118* and *MYB115* can promote the transition from asexual development to embryo formation, independent of WUSCHEL (WUS) signaling [70]. WRKY TFs are also involved in the SE process of *Panax ginseng* [71]. AP2/ERF TFs are essential for seed development, organ morphology, and other aspects of plant development by responding to ethylene, cytokinin, and auxin signaling [72]. Piyanuch Piyatrakul and colleagues highlighted the significant regulatory role of the AP2/ERF superfamily in SE [73]. Among the AP2/ERF family, the *BABY BOOM/PLETHORA4* (*BBM/PLT4*) genes are key regulators in the plant embryo development process. In *Arabidopsis*, *BBM* plays a crucial role during both embryo and endosperm development [74]. *ENHANCER OF SHOOT REGENERATION1* (*ESR1*) is induced by cytokinins and regulates the shoot regeneration process in *Arabidopsis* [75]. Several members of the AP2 family play crucial roles in plant embryogenesis and organ development [76]. The NF-Y TF family binds to the CCAAT box and regulates various developmental processes [77]. Early studies have shown that NF-YB9 plays a significant role in both early and late stages of embryo development, regulating processes such as embryo cell fate, cotyledon development, and embryo maturation [78]. As research progresses, it has become clear that other members, such as NF-YB6, NF-YA3, and NF-YA8 also play critical roles in embryogenesis [79]. In the MADS TF family, AGAMOUS-Like 15 (AGL15) and AGL18 are key regulators in the plant embryo development process. AGL15 directly targets key regulatory factors in embryogenesis, such as the *AFL* (*ABI3/FUS3/LEC2*) genes [80]. Auxin Response Factors (ARFs) are TFs that regulate the expression of auxin-responsive genes and play a central role in auxin signaling. These factors specifically bind to auxin response elements (AREs) to either promote or inhibit gene expression [81]. In the process of zygotic embryo formation in wheat, four key transcription factor families—TCPs, ARFs, MYBs, and WOXs—are involved in a core transcriptional regulatory network. This network includes a regulatory module comprising LEC1-MYB118-ZHD5-LEC2-BBM, which plays a central role in embryo development [82]. Dlo019079 (SCL3) and Dlo027427 (MOF1) were annotated to be involved in plant hormone signal transduction. Since plant hormone signaling is crucial in the process of somatic embryogenesis in plants [83], it is suggested that TFs annotated to the plant hormone signal transduction pathway may play an important role in DIMBOA-induced somatic embryogenesis. Hence, DIMBOA likely participates in the early SE process of longan by regulating the expression of these crucial TFs. By modulating the expression levels of TFs, such as AP2, NF-Ys, ARFs, MYB, WRKY, and MADS-box, etc., DIMBOA influences various aspects of the embryonic development process, including cell fate, organ development, and auxin signaling, all of which are essential for proper SE induction.

### 3.3. DIMBOA May Regulate the Morphogenesis of Early SE in Longan by Affecting the VB_6_ Metabolic Pathway

VB_6_ is a collective term for a group of six interconvertible compounds: PN, PL, PM and their phosphorylated derivatives. VB_6_ plays an essential role as a cofactor in a range of biochemical reactions. ROS are highly reactive molecules that play a dual role in plants. While they are necessary for several physiological processes, excessive ROS accumulation due to abiotic stresses, such as drought and osmotic stress, can lead to oxidative damage [84]. The major types of ROS in plants include singlet oxygen (^1^O_2_), hydroxyl radical (OH^−^), superoxide anion (O_2_^−^), and hydrogen peroxide (H_2_O_2_). VB_6_, particularly its active form pyridoxal phosphate (PLP), has been shown to have antioxidant properties. It can quench ROS and protect plants from oxidative damage [85]. Research has demonstrated that exogenous VB_6_ can protect plant protoplasts from cell death induced by ^1^O_2_, which is a highly reactive form of ROS [86]. The *PDX1* gene, essential for VB_6_ biosynthesis, is expressed in all plant tissues and plays a critical role in managing oxidative stress. Studies have shown that mutants with defective *PDX1*, such as *pdx1*, exhibit increased sensitivity to osmotic and oxidative stress, highlighting the importance of VB_6_ in ROS scavenging [87]. In maize, a mutant called “*small kernel 2*” (*smk2*), which is deficient in VB_6_, shows high sensitivity to salt stress. However, applying exogenous PN and PLP has been found to reduce ROS accumulation in the roots and improve the plant’s salt tolerance. This suggests that VB_6_ helps to balance ROS levels and ABA in plants, contributing to stress tolerance [88]. VB_6_’s pyridine ring contains hydroxyl and amine groups, which can react directly with ROS radicals, acting as an effective antioxidant. Therefore, VB_6_ plays a significant role in regulating ROS levels in plants under stress conditions. DIMBOA, an exogenous compound, has also been shown to influence ROS accumulation in plants. For example, light-induced stimulation in maize triggers the production of benzoxazinoid (BX) compounds, which leads to an increase in H_2_O_2_ content on the light-exposed side of the coleoptile [26]. In oats, exogenous DIMBOA treatment affects the activity of peroxidases, promoting H_2_O_2_ synthesis [20]. Similarly, when coumarin and DIMBOA are applied together to alfalfa seedlings, they reduce ROS accumulation while increasing the activities of CAT, POD, APX, and ASA, thus enhancing the seedlings’ antioxidant defense capacity [17]. These findings suggest that DIMBOA modulates ROS levels in plants, which may further contribute to stress tolerance and cellular protection. In conclusion, both DIMBOA and VB_6_ can regulate ROS levels in plants.

H_2_O_2_ plays a crucial role in the regulation of plant SE and has been shown to influence the efficiency of SE in various plant species. In systems like larch somatic embryogenesis, reducing endogenous H_2_O_2_ levels has been found to improve embryogenesis efficiency, suggesting that an optimal level of H_2_O_2_ is required for successful SE [89]. In tangerine somatic embryo induction, the H_2_O_2_ content gradually increases at the beginning of the process and then decreases as the embryos progress through development [90]. This pattern indicates that higher ROS levels, including H_2_O_2_, are required during the early stages of embryo formation, possibly to promote cellular differentiation and the transition from callus to embryo. Similarly, in banana somatic embryogenesis, H_2_O_2_ levels rise as the callus tissue develops into globular embryos, and then decrease during the transition from globular to mature embryo stages [91]. These changes in H_2_O_2_ concentration suggest that ROS, including H_2_O_2_, play a role in regulating different stages of embryo development. In flower elm, during the SE process, the H_2_O_2_ content, along with the activities of enzymes such as polyphenol oxidase (PPO), superoxide dismutase (SOD), ascorbate peroxidase (APX), peroxidase (POD), and catalase (CAT), is significantly higher in EC compared to NEC [92]. Low levels of H_2_O_2_ and enzyme activity are detrimental to SE induction [92], highlighting the importance of ROS in driving the somatic embryogenesis process. These studies demonstrate that the accumulation of H_2_O_2_ and regulation of ROS levels are critical factors that influence the success of somatic embryogenesis. An appropriate balance of ROS, including H_2_O_2_, is necessary to stimulate the transition from callus formation to the development of somatic embryos. Therefore, ROS, especially H_2_O_2_, are vital regulators of plant embryo formation, and managing their levels can enhance the efficiency of SE in plant tissue culture systems.

**Figure 12:**
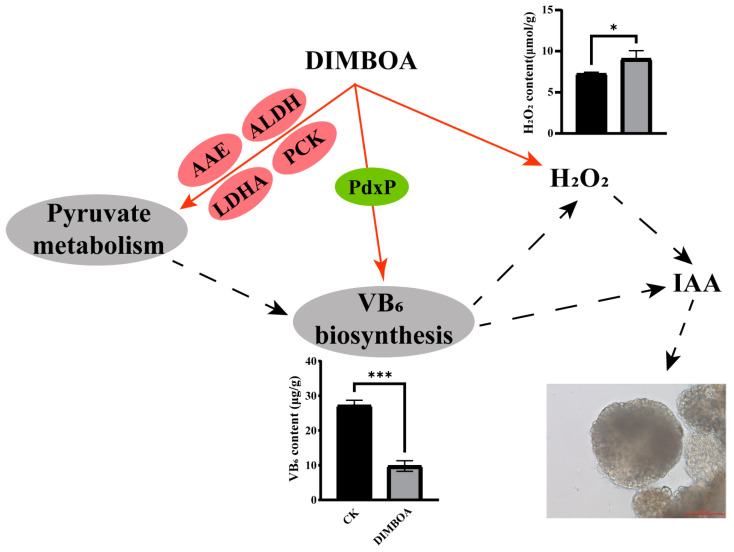
Schematic diagram of the possible molecular mechanisms by which DIMBOA promotes early somatic embryogenesis in longan. The gray ellipse represents the KEGG pathway enriched in this study, the red ellipse represents the DEGs that are upregulated, the green ellipse is the DEGs that are downregulated, the solid red arrows indicate the results of this study, and the dashed arrows indicate the reported results. CK is an abbreviation for the check. An asterisk indicates distinctiveness (* *p* ≤ 0.05 and *** *p* ≤ 0.001).

A crucial phytohormone that controls SE in plants is auxin. According to a large number of studies, somatic embryo development can be induced by ectopic expression of TFs that are typically expressed in embryos as well as by exogenous auxin treatments. The crucial role that auxin performs in the process of somatic embryogenesis becomes apparent by the fact that these pathways eventually activate by encouraging auxin biosynthesis and signaling. VB_6_ is an essential cofactor for several enzymes involved in important biochemical reactions in plants, including those involved in the biosynthesis of auxin. Specifically, VB_6_ can regulate the production of endogenous hormones such as IAA, which is crucial for plant growth, development, and metabolism [93]. The biosynthesis of the major auxin, IAA, primarily follows the tryptophan (Trp)-dependent pathway, which requires PLP, the active form of VB_6_, as a coenzyme for certain enzymes involved in this pathway [94,95]. In VB_6_-deficient mutants, such as the *pdx1* mutant, the local biosynthesis of auxin in the roots is impaired, leading to reduced auxin levels at the root apex and stunted growth [96]. Supplementation of exogenous VB_6_ (e.g., pyridoxine HCl) has been shown to promote IAA biosynthesis and significantly increase endogenous IAA content in plants like maize [93]. Deficiency in VB_6_ biosynthesis disrupts the homeostasis of essential plant hormones like IAA and ethylene, leading to defects in growth and development [97]. Knockout of the *PDX2* gene or simultaneous knockout of the *PDX1.1* and *PDX1.3* homologs has been found to be lethal in certain plant species, halting embryo development at the globular stage. Additionally, ROS, including H_2_O_2_, are known to interfere with auxin transport and the regulation of cell proliferation and differentiation in roots [98,99]. Recent research has shown that H_2_O_2_ can modulate auxin’s polar transport, thus influencing the accumulation and redistribution of auxin in plants, while ROS primarily suppress auxin signaling to regulate growth and development [27]. Overall, VB_6_ and its role in regulating auxin biosynthesis, along with the influence of ROS on auxin transport and signaling, are critical factors in regulating somatic embryogenesis. Proper auxin synthesis and signaling are essential for the successful initiation and development of somatic embryos in plants. In this study, qPCR analysis revealed that the expression of key genes involved in IAA synthesis was significantly downregulated. Both the VB_6_ biosynthesis pathway and the pyruvate metabolism pathway were significantly enriched under DIMBOA treatment, with the VB_6_ biosynthesis pathway showing the most prominent enrichment. In the VB_6_ biosynthesis pathway, *PdxP* (Dlo006333 and Dlo006334) are critical genes for VB_6_ synthesis. Both genes were significantly downregulated under DIMBOA treatment, leading to a significant reduction in endogenous VB_6_ content. Additionally, under DIMBOA treatment, DEGs in the pyruvate metabolism pathway showed significant upregulation, which might reduce the pyruvate content in longan embryogenic cultures. It is known that D-erythrose-4-phosphate, glyceraldehyde-3-phosphate, and pyruvate are precursors for the de novo biosynthesis of PLP, the bioactive form of VB_6_ [36]. The reduction in pyruvate content would, therefore, impact the biosynthesis of VB_6_. In conclusion, exogenous DIMBOA may affect H_2_O_2_ by regulating the expression of key genes involved in VB_6_ biosynthesis and altering the accumulation of precursor substances (e.g., pyruvate) to reduce VB_6_ levels, or by directly affecting H_2_O_2_ levels, both of which affect endogenous IAA levels, and further influence the morphogenesis of the early somatic embryos of longan (Figure 12).

## 4. Materials and Methods

### 4.1. Plant Material

The experimental materials were taken from the EC of ’honghezi’ longan cultivated by the Institute of Horticultural Plant Bioengineering of Fujian Agriculture and Forestry University, and cultured with reference to Lai Zhongxiong’s culture method. The 20-day-old light yellow longan ECs were transferred to an MS-based medium supplemented with 2% sucrose and 7 g/L agar, and treated with 0 (the check, CK) and 0.1 mg/L DIMBOA. Samples were taken for weighing 9 days after treatment. The samples were immediately snap-frozen in liquid nitrogen and stored at −80 °C for subsequent analysis. The experiment was carried out with three biological replicates.

### 4.2. Transcriptome Library Construction and Sequencing

Based on previous experimental findings, it was found that treatment with 0.1 mg/L DIMBOA significantly promoted the early somatic embryogenesis of longan, and had the most significant effect on the accumulation of endogenous IAA content [25]. Furthermore, 9 days after treatment, the morphological structure of embryogenic cultures showed the most distinct differences between the check and 0.1 mg/L DIMBOA-treated groups. Therefore, embryogenic cultures treated with 0 and 0.1 mg/L DIMBOA for 9 days were chosen as materials for relative transcriptome sequencing analysis in this study. The control group samples were labeled check1, check2, and check3, and the treatment group samples were labeled D1, D2, and D3, resulting in a total of six mRNA libraries. Biomarker Biotechnology Corporation extracted total RNA from the six groups of longan embryogenic cultures. The purity and concentration of RNA were assessed using a NanoDrop 2000 spectrophotometer, and RNA integrity was verified using the Agilent 2100/LabChip GX. After samples passed quality control, library construction and mRNA transcriptome sequencing were carried out.

HISAT2 [100] (Hierarchical Indexing for Spliced Alignment of Transcripts) software was used to quickly and accurately compare the clean reads with the reference genome, and to acquire aligning information for the reads on the reference genome. The reads were then assembled using StringTie [101] to reconstruct the transcriptome for subsequent analysis.

### 4.3. Analysis of Differentially Expressed Genes

Gene expression was normalized using FPKM [102] (fragments per kilobase of transcript per million fragments mapped) using StringTie via a maximum flux algorithm as a measure of transcript or gene expression level. DEG (differentially expressed gene) analysis was conducted using DESeq2 [103] software, with the thresholds of |log2(fold change)| > 1 and FDR < 0.01 for filtering DEGs. GO (Gene Ontology) enrichment analysis was carried out using the topGO software. DEGs were mapped to GO terms in the GO database, and the number of genes associated with each term was calculated to generate a target gene list for each GO function. The hypergeometric test was used to identify significantly enriched GO terms, providing insights into the main biological functions of the DEGs. GO (Gene Ontology) enrichment analysis was carried out using the topGO software. DEGs were mapped to GO terms in the GO database, and the number of genes related to each term was calculated to generate a target gene list for each GO function. The hypergeometric test was used to identify significantly enriched GO terms, providing insights into the main biological functions of the DEGs. For KEGG (Kyoto Encyclopedia of Genes and Genomes) pathway enrichment analysis, DEGs were analyzed to identify pathways significantly deprived proportional to the entire genome background using the hypergeometric test. The top 20 significantly enriched pathways, based on the smallest q-values, were visualized using bubble plots generated by ClusterProfiler.

### 4.4. Determination of H_2_O_2_ Content in Embryogenic Cultures of Longan

The hydrogen peroxide content in longan embryogenic cultures was measured using a hydrogen peroxide assay kit purchased from Heruibio. First, 0.1 g of longan embryogenic culture was weighed and mixed with 1 mL of reagent I. The mixture was ground on ice to a homogenous slurry and then transferred to an EP tube. The sample was centrifuged at 8000× *g* and 4 °C for 10 min, and the supernatant was collected and kept on ice until measurement. Finally, following the kit instructions, the reagent was added to the supernatant in the EP tube, and after standing at room temperature for 5 min, absorbance at 415 nm was measured using a microplate reader. The hydrogen peroxide content (μmol/g) was calculated using the formula: H_2_O_2_ content (μmol/g) = [(∆A − 0.0006) ÷ 0.3744 × V1] ÷ (W × V1 ÷ V2). V1 is the volume of sample added to the reaction system, V2 is the volume of extract solution added, and W is the sample weight (g).

### 4.5. Determination of VB_6_ Content in Embryogenic Cultures of Longan

VB_6_ content in longan embryogenic cultures was measured using a VB_6_ assay kit purchased from Suzhou Keming Biotechnology Co., Ltd., Suzhou, China. First, 0.1 g of longan embryogenic culture, which had been stored at −80 °C, was weighed and mixed with 0.6 mL of extraction solution. The mixture was ground into a homogenous slurry. Then, it was incubated at 60 °C for 30 min, followed by the addition of 0.4 mL of distilled water. The solution was mixed thoroughly and centrifuged at 16,000 rpm at 25 °C for 10 min. The supernatant was collected for measurement. During the measurement, the blank was set to zero using distilled water, and absorbance at 390 nm was recorded as A_blank and A_sample. The change in absorbance (∆A) was calculated as ∆A = A_sample-A_blank. The standard curve was y = 0.3635x + 0.0205, R2 = 0.9986. The VB_6_ content (μg/g) was calculated using the formula VB_6_ content (μg/g) = 13.76 × (∆A − 0.0205)/W.

### 4.6. Quantitative PCR

In previous studies, based on second-generation genome data, transcriptome sequencing analysis of early somatic embryogenesis (SE) in longan revealed that key genes in the IAA biosynthesis pathway, including *YUCCA* (Dlo_013505.1, Dlo_027234.1, and Dlo_024140.1), *AAO1* (Dlo_016663.1), *NIT* (Dlo_032471.1), and *ST5a* (Dlo_007467.1), were significantly expressed during early SE in longan [2]. In this study, we used longan’s third-generation genome data to perform a BLAST search and selected 6 key genes involved in IAA biosynthesis and ROS-related genes (*SOD*, *POD*, *CAT*, and *MSD*) for qPCR analysis following exogenous DIMBOA treatment. These genes included *YUCCA* (Dlo000667, Dlo002659, Dlo012198), *AAO1* (Dlo027804), *NIT* (Dlo025950), and *ST5a* (Dlo016797). Additionally, key genes were selected based on transcriptome sequencing data for qPCR validation. Total RNA was extracted from all samples using TransZol Up (TRANS), and RNA concentrations were measured using a Nanodrop 2000 spectrophotometer (Thermo Scientific, Wilmington, DE, USA). cDNA synthesis was performed according to the Hifair^®^1st Strand cDNA Synthesis kit (Yeasen, Shanghai, China) kit. EF-1ɑ was used as the internal reference gene for qPCR analysis with the single-reference method. cDNA was diluted 10-fold and used as a template for amplification. qRT-PCR was performed using a Roche LightCycler 96 instrument. The specificity of primers for longan BX biosynthesis key genes was checked using DNAMAN 2.0. Gene expression was quantified using the 2^−△△CT^ method. Statistical analysis of the relative expression levels was performed using IBM SPSS Statistics 26, and three technical replicates were set up for each qRT-PCR experiment. All primers used in this study are listed in Supplementary Table S5.

## 5. Conclusions

In this study, longan embryogenic cultures were treated with 0 mg/L DIMBOA (control group, check) and 0.1 mg/L DIMBOA (treatment group, D) for transcriptomic sequencing. A total of 478 DEGs were detected between check vs. D, with 193 genes upregulated and 285 genes downregulated. GO analysis revealed that five GO terms were significantly altered under DIMBOA treatment: cellular response to phosphate starvation, heme binding, peroxidase activity, pectin catabolic process, and response to oxidative stress. KEGG pathway analysis demonstrated that the DEGs were mainly enriched in the VB_6_ metabolic pathway, phenylpropanoid-related pathways, and carbohydrate metabolic pathways. Moreover, TFs such as MYB, ZF, bHLH, LBD, NAC, and WRKY are likely involved in DIMBOA-induced early SE in longan. qPCR results indicated that the expression levels of key genes involved in endogenous IAA biosynthesis were downregulated under external DIMBOA treatment, suggesting that DIMBOA might influence the endogenous IAA levels in longan embryogenic cultures. DIMBOA might regulate VB_6_ biosynthesis to reduce VB_6_ content, which might indirectly affect H_2_O_2_ levels, or it may directly impact H_2_O_2_ levels, further influencing endogenous IAA levels. Ultimately, these effects could contribute to the promotion of early somatic embryo morphological development in longan.

## Data Availability

The datasets presented in this study have been submitted to the NCBI database but have not yet been released.

## Supplementary Materials

The following supporting information can be downloaded at https://www.mdpi.com/article/10.3390/plants14030442/s1. Table S1: The information of the top 20 KEGG pathways; Table S2: The information of the top 20 KEGG pathways; Table S3: The FPKM of DEGs in this study; Table S4: Alternative splicing event count; Table S5: Primers used for qRT-PCR analysis.
